# Individuals Infected with SARS-CoV-2 Prior to COVID-19 Vaccination Maintain Vaccine-Induced RBD-Specific Antibody Levels and Viral Neutralization Activity for One Year

**DOI:** 10.3390/v17050640

**Published:** 2025-04-29

**Authors:** Christina S. Mcconney, Devin Kenney, Christina S. Ennis, Erika L. Smith-Mahoney, Maria Jose Ayuso, Jiabao Zhong, Florian Douam, Manish Sagar, Jennifer E. Snyder-Cappione

**Affiliations:** 1 Department of Virology, Immunology, and Microbiology, Chobanian & Avedisian School of Medicine, Boston University, Boston, MA 02118, USA; csmcc@bu.edu (C.S.M.); kenneydj@bu.edu (D.K.); mayuso421@gmail.com (M.J.A.); jbzhong@bu.edu (J.Z.); fdouam@bu.edu (F.D.); 2 National Emerging Infectious Diseases Laboratories, Boston University, Boston, MA 02118, USA; 3 Cancer Center, Chobanian & Avedisian School of Medicine, Boston University, Boston, MA 02118, USA; ennisc@bu.edu; 4 Department of Medicine, Chobanian & Avedisian School of Medicine, Boston University, Boston, MA 02118, USA; manish.sagar@bmc.org

**Keywords:** severe acute respiratory syndrome coronavirus 2 (SARS-CoV-2), nucleocapsid protein, receptor-binding domain (RBD), hybrid immunity, longitudinal, neutralization activity

## Abstract

The effectiveness of multiple COVID-19 vaccinations in individuals with a history of SARS-CoV-2 infection remains unclear; specifically, elucidation of the durability of anti-viral antibody responses could provide important insights for epidemiological applications. We utilized the BU ELISA protocol to measure the circulating SARS-CoV-2 receptor-binding domain (RBD) and nucleocapsid (N) specific IgG and IgA antibody levels in a cohort of individuals infected with SARS-CoV-2 in the spring of 2020, with the sample collection spanning six months to two years post-symptom onset. Further, we interrogated the neutralization activity of these samples against the ancestral SARS-CoV-2 (WA-1) and Delta and Omicron (BA.1) variants. Consistent with previous studies, we found a more rapid waning of anti-N compared to anti-RBD antibodies in months prior to the first vaccinations. Vaccine-induced antibody responses in individuals previously infected with SARS-CoV-2 were elevated and sustained for more than one year post-vaccination. Similarly, neutralization activity against WA-1, Delta, and Omicron increased and remained higher than pre-vaccination levels for one year after the first COVID-19 vaccine dose. Collectively, these results indicate that infection followed by vaccination yields robust antibody responses against SARS-CoV-2 that endure for one year. These results suggest that an annual booster would stably boost anti-SARS-CoV-2 antibody responses, preventing infection and disease.

## 1. Introduction

As we enter the sixth year since the start of the COVID-19 pandemic, questions surrounding the durability of hybrid immunity—immune protection resulting from the combination of at least one COVID-19 vaccine and one historical SARS-CoV-2 infection—remain. Tragic outcomes from acute COVID-19 have decreased considerably since 2020, as the establishment of effective vaccines coupled with widespread infection history have resulted in much of the US population benefiting from hybrid immunity [1,2]. Initial mRNA vaccine trial results showed an excellent induction of antibodies with more than 90% vaccine efficacy (prevention of infection); however, this sterilizing immunity waned within six months, and breakthrough infections occurred [3,4,5]. To date, the most durable and protective immune responses to SARS-CoV-2 were found in those with a history of both infection and COVID-19 vaccination [6,7,8].

While numerous studies have measured anti-SARS-CoV-2 antibodies, readouts from early studies predominantly did not span more than a few months post-infection or post-vaccination [9,10,11,12,13,14]. More recently, the long-term durability of SARS-CoV-2-specific antibodies has been reported, with sustained responses after 8–12 months [9,15]. Also, comparisons of anti-SARS-CoV-2-spike (S) and anti-nucleocapsid (N) antibody level trajectories after infection have shown longer-lasting anti-S than anti-N IgG levels [11,16,17]. Plasma SARS-CoV-2 neutralizing activity against the SARS-CoV-2 ancestral, Delta, and Omicron variants, are high post-infection and/or vaccination but wane early, within 3–6 months [18,19]. Additionally, while neutralization assays are the current “gold standard” for correlates of protection (CoPs) from SARS-CoV-2 infection, evidence for other CoP candidates, such as serum IgG and IgA levels, has accumulated [12,15,16,20,21,22].

When compared to only SARS-CoV-2 infection or COVID-19 vaccination, hybrid immunity results in the most significant increase in antibody titers, up to 3.6-fold higher than increases induced by vaccination alone [23]. Additionally, antibodies in hybrid immune individuals demonstrated broader neutralization activity against multiple SARS-CoV-2 variants [24]. Until recently [25,26,27], the longevity of these antibodies had not been interrogated over a year post-vaccination or infection, and the endurance of viral neutralization activity has not been clearly established over a year post-vaccination.

In this post-pandemic era, the concern now shifts to understanding the longevity and effectiveness of the predominant hybrid immunity and how the receipt of additional COVID-19 vaccines coupled with previously acquired SARS-CoV-2 infection-induced immune memory may minimize the severity of illness and viral spread and thereby guide vaccination schedules. We measured the long-term stability of anti-N and anti-S (specifically the receptor-binding domain, RBD) IgG and IgA after vaccination in previously infected individuals and determined how these antibody levels tracked with SARS-CoV-2 neutralization activity for an extended period of time (up to two years) post-infection. By measuring plasma levels of anti-N and anti-RBD IgG and IgA in tandem with neutralization data, this study aims to better inform the relationship between neutralization activity and circulating antibody levels in relevant long-term time frames. We used a sensitive enzyme-linked immunosorbent assay protocol, the BU ELISA [28], and SARS-CoV-2 viral neutralization assays to measure the amount of anti-SARS-CoV-2 IgG and IgA antibodies and neutralization activity from a cohort (n = 46) of patients enrolled at the Boston-based prospective clinical biorepository, the Mass General Brigham (MGB) Biobank, spanning from early 2020 to mid-to-late 2022. This body of work contributes to the growing evidence demonstrating the longevity of anti-SARS-CoV-2 antibodies in time frames that extend beyond a year post-infection and/or vaccination [9,23,29].

## 2. Materials and Methods

### 2.1. Study Population

Plasma samples from SARS-CoV-2-positive patients were obtained through the MGB Biobank, comprising 46 patients recruited to either the outpatient clinic or inpatient wards beginning in March of 2020. Plasma was drawn beginning in acute disease (within 10 days of symptom onset) and continued intermittently for an additional 4–25 months (median, 13.7) for a total of three to eight plasma samples per patient. Inclusion criteria for patients included a confirmed PCR positive test for SARS-CoV-2 and at least two longitudinal plasma samples collected post-symptom onset. When vaccines became available, sample collection for 12 subjects continued; criteria for continued inclusion in this part of this study included samples from at least two time points after vaccination (median, 24.3 (21.4–25.4) months post-symptom onset (PSO)). These 12 subjects completed the primary one-dose (Johnson & Johnson vaccine administered, n = 1) or two-dose (Moderna or Pfizer administered, n = 11) vaccine series and 10/12 (83%) of the subjects received a booster during the course of this study. In all cases, patients who are identified as “Inpatient (IP)” or “Outpatient (OP)” refer to whether they required hospitalization during acute illness. All samples were collected beginning in acute illness through post-recovery. Any subsequent fluctuations in anti-N antibody levels represent suspected reinfection cases with SARS-CoV-2 or an endemic coronavirus (not PCR confirmed).

### 2.2. Ethics Statement

Human subject review and permissions for this cohort were obtained from the Boston University Chobanian and Avedisian School of Medicine’s Institutional Review Board. Samples and clinical data were deidentified upon sample collection from the MGB Biobank.

### 2.3. SARS-CoV-2 Antiviral Antibody Measurements

Antibodies reactive to SARS-CoV-2 RBD (a gift from the Schmidt lab at the Ragon Institute of MGB, MIT, and Harvard, Cambridge, MA, USA), Nucleocapsid-His recombinant Protein (N) (Cat #40588-V08B, Sino Biological, Beijing, China), EBV Glycoprotein gp350 (Cat# 40373-V08H, Sino Biological, Beijing, China), CMV (Cat# CM-217, ProSpec, Rehovot, Israel), or Influenza A H1N1 Hemagglutinin/HA (Cat# 11085-V08B, Sino Biological, Beijing, China) were assayed from plasma using the BU ELISA (Yuen et al., 2021 [28]). Briefly, wells of 96-well plates (Cat# 15041, Thermo Fisher, Waltham, MA, USA) were coated with 50 µL/well of 2 mg/mL or 1 mg/mL of each respective protein in sterile PBS for one hour at room temperature. The coating solution was removed, and the plate was washed three times with 200 µL/well of sterile PBS. The plate was blocked with 200 µL/well of casein blocking solution (Cat# 37528, Thermo Fisher, Waltham, MA, USA) for one hour at room temperature and then washed again as described. Plasma samples, diluted 1:100 or 1:500 in casein blocking solution, were added to the plate at 50 µL/well. Samples were run as singlets. Dilution buffer alone was added to blank wells and the plate was incubated for one hour at room temperature. Diluted samples were removed, and the plate was washed three times with 0.05% PBS-Tween 20 (PBS-Tween). Immediately after, anti-human horseradish peroxidase-conjugated secondary antibodies for IgG (Cat# A18817, Thermo Fisher, Waltham, MA, USA) and IgA (Cat# 109-035-011, Jackson ImmunoResearch, West Grove, PA, USA) diluted 1:2000 in casein, were added to all wells at 50 µL/well and incubated for 30 min at room temperature. Finally, the plates were washed four times with PBS-Tween, and, then, 3,3′,5,5′-Tetramethylbenzidine (TMB)-ELISA substrate solution (Cat# 34028, Thermo Fisher, Waltham, MA, USA) was added to all wells at 50 µL/well and incubated in the dark. Incubation occurred until a difference in color between the seventh dilution (~1.4 ng/mL) and the diluent-only ‘zero’ well was visualized (8–30 min). Once a difference was observed, ELISA stopping solution for TMB was added at 50 µL/well, and optical density was measured at 45 nm (OD 450 nm) on a SpectraMax190 Microplate Reader. Values that fell below the seventh dilution (~1.4 ng/mL) were determined to be below our limit of detection (LOD).

### 2.4. Cell Lines

VeroE6 cells were grown in Dulbecco’s modified Eagle’s medium (DMEM) supplemented with 10% heat-inactivated fetal bovine serum (Bio-Techne, R&D systems, Minneapolis, MN, USA) and 1% (*v*/*v*) Penicillin Streptomycin (P/S) (Thermo Scientific, Waltham, MA, USA). A549-hACE2/hTMPRSS2 and Caco-2-hACE2/hTMPRSS2 were grown in DMEM with 10% FBS 1% P/S and were supplemented with puromycin and blasticidin selection antibiotics.

### 2.5. Generation of SARS-CoV-2 Isolates

All replication-competent SARS-CoV-2 experiments were performed in a BSL-3 facility at the Boston University National Emerging Infectious Diseases Laboratories. SARS-CoV-2 WA-1 clinical isolate (2019-nCoV/USA-WA1/2020 strain; NCBI accession number: MN985325) was obtained from BEI Resources (Manassas, VA, USA). SARS-CoV-2 Delta (B.1.617.2) and Omicron (BA.1) were generously provided by the laboratories of Drs. John Connor and Mohsan Saeed (National Emerging Infectious Diseases Laboratories, Boston University, Boston, MA, USA). Growth and titering of SARS-CoV-2 WA-1 and Delta by isolates were performed on VeroE6 cells. Growth and titering of SARS-CoV-2 Omicron BA.1 was performed on Caco-2 cells expressing hACE2/hTMPRSS2. Briefly, 1 × 10^7^ cells were plated in a T-175 flask one day prior to virus generation. The next day, cells were infected with virus diluted in 10 mL of Opti-MEM (Thermo Fisher Scientific, Waltham, MA, USA) and incubated for one hour at 37 °C to allow for virus adsorption. After incubation, 15 mL of DMEM containing 10% FBS and 1% penicillin/streptomycin were added to cells and incubated overnight at 37 °C with 5% CO_2_. The next day, medium was removed, cells were rinsed with 1× PBS, and 25 mL of fresh DMEM containing 2% FBS was added. Cells were assessed for cytopathic effect (CPE) and, after signs of significant CPE, medium was collected, filtered with a 0.22 μm filter, and concentrated by sucrose gradient (WA-1 and Delta) or PEG6000 (Omicron BA.1). Concentrated virus was suspended in sterile 1× PBS, aliquoted, and stored at −80 °C.

### 2.6. Serum Neutralization Assays

One day prior to the experiment, 2 × 10^4^ A549-hACE2/hTMPRSS2 expressing cells were plated in 96-well plates. Prior to dilution, serum was decomplemented at 56 °C for 30 min, and an initial dilution of 1:5 was prepared in OptiMEM. Two-fold dilutions were subsequently prepared and mixed with SARS-CoV-2 WA-1, Delta (clinical isolate), or Omicron BA.1 (clinical isolate) for one hour at room temperature and then plated onto cells. Viral adsorption was performed at 37 °C for one hour. The serum/virus mix was then removed, and 200 μL of fresh DMEM containing 2% FBS and 1% penicillin/streptomycin were added. Cells were incubated for 24 h at 37 °C with 5% CO_2_. Cells were then fixed with 10% formalin for one hour before immunofluorescence staining with an anti-SARS-CoV-2 N antibody. Quantification of infection was performed by image capture with a Biotek Cytation imager (Agilent, Santa Clara, CA, USA) and analyzed using Cell Profiler (version 4.0.7).

### 2.7. Immunofluorescence Staining for SARS-CoV-2 N Protein

Fixed cells were washed twice with 1× PBS and then permeabilized with 1× PBS + 0.1% Triton-X (PBST) for 30 min at room temperature. After permeabilization, cells were blocked in PBST + 10% goat serum (*v*/*v*) and 1% BSA (*w*/*v*) for 30 min at room temperature before overnight incubation at 4 °C with rabbit polyclonal anti-SARS-CoV nucleocapsid antibody (1:2000 dilution; Rockland, Limerick, PA, USA, Cat #200-401-MS4). After primary antibody incubation, the cells were washed three times and stained with Alexa568-conjugated goat anti-rabbit antibody (1:1000 dilution; Thermo Fisher, Cat #A11011) for one hour protected from light at room temperature. Cells were washed three times with 1× PBS and counterstained with Hoechst.

### 2.8. Data Visualization and Statistics

Statistical analyses were performed using GraphPad Prism v10.3.0 (461) (GraphPad Software, San Diego, CA, USA) and R Statistical Software (4.4.1, R core team 2024).

#### 2.8.1. Calculation of Average Slope of Antibody Levels over Time

Slopes across visits for circulating anti-RBD (IgG and IgA) and -N (IgG and IgA) were calculated per individual for three specified time frames: 0–260 days PSO, after vaccination, and 0–495 days PSO, using the Theil–Sen slope estimator. Slopes per individual were averaged to generate one average slope. Statistical significance of the calculated average slope was assessed by comparing it to a null hypothesis (slope = 0) using the Wilcoxon signed rank test. Average slopes for virus-specific neutralization activity were calculated in an identical manner for the following specified intervals: “before [vaccination] vs. last [time point]”, “after [vaccination] vs. last [time point]”, and “before [vaccination] vs. after [vaccination]”. A two-sided *p*-value of <0.05 was considered statistically significant.

#### 2.8.2. Calculation of Overall Changes in Circulating Antibody Levels

**Figure 1:**
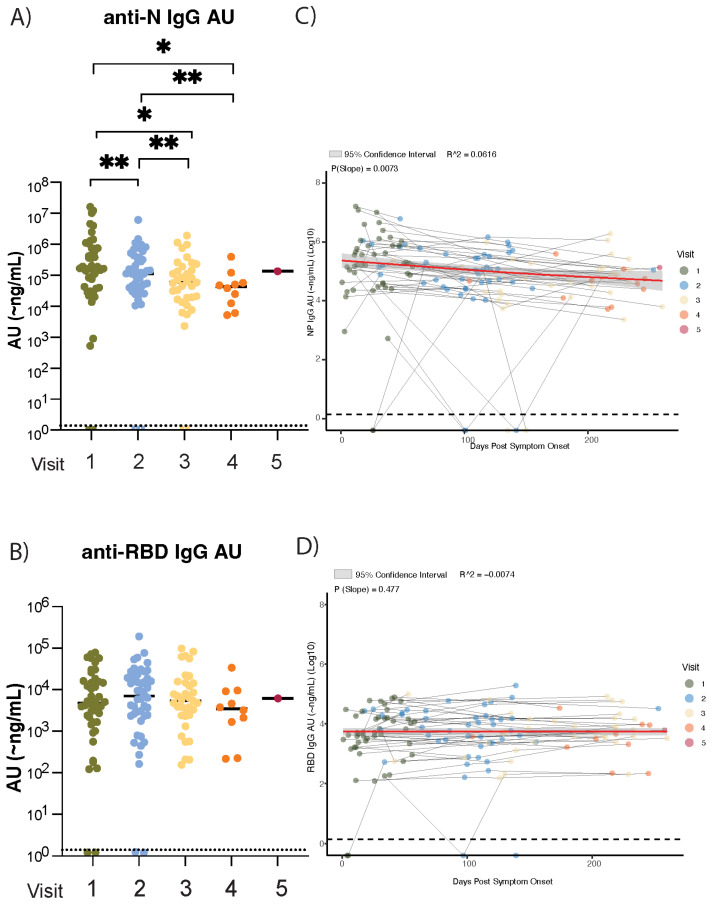
Anti-N and anti-RBD IgG levels in SARS-CoV-2 individuals up to nine months post-COVID-19 symptom onset. Scatter plots showing the levels (~ng/mL) of circulating (**A**) anti-N IgG and (**B**) anti-RBD IgG antibodies measured at each clinic visit per individual (colors indicate visit number) post-PCR-positive SARS-CoV-2 diagnosis (n = 46), pre-vaccination. Statistical significance in circulating antibody levels between visits is determined using Wilcoxon matched-pair signed rank tests (**A**,**B**). Lack of an asterisk indicates a lack of significance. Antibody trajectories per individual (**C**,**D**) are shown with a generalized additive model (gam) trend line (red) and 95% CI. The average slope across all individuals for circulating anti-N and anti-RBD IgG is calculated using the Theil–Sen slope estimator. Statistical significance is assessed with the Wilcoxon-matched signed rank test (see Methods). Wilcoxon signed rank tests are performed to determine changes in the overall slope of circulating antibody levels, with the day of each person’s symptom onset set as time point 0. *p* > 0.1234 (non-significant), *p* < 0.0332 (*), *p* < 0.0021 (**). Dashed lines represent the LOD of the BU ELISA (LOD = ~1.4 ng/mL).

**Figure 2:**
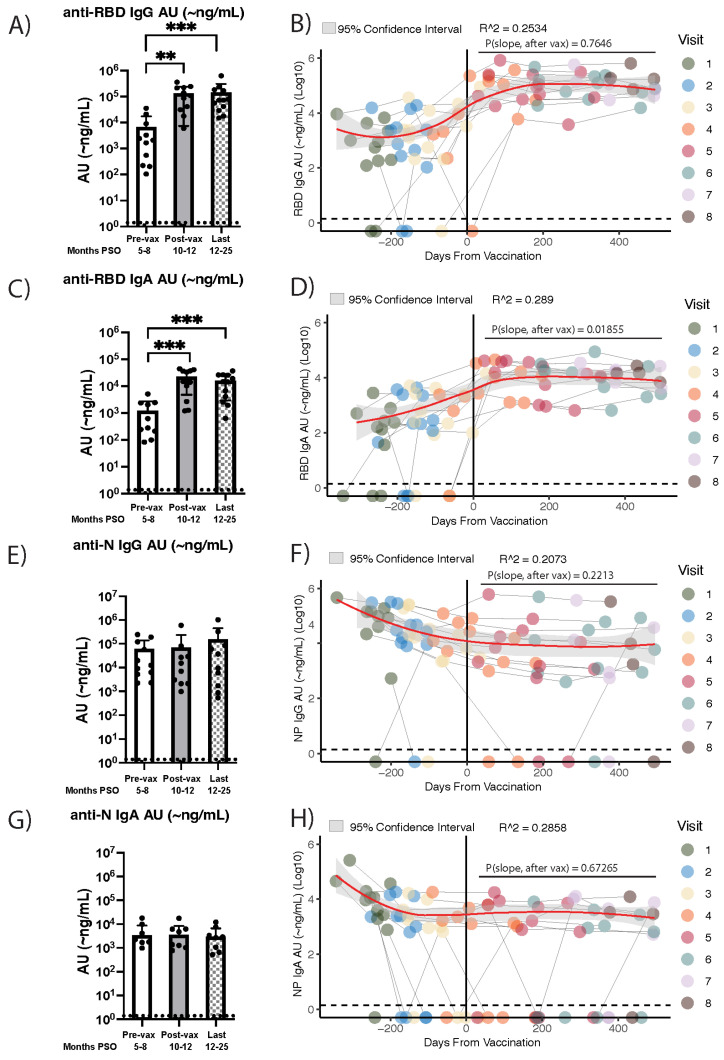
Results of the impact of COVID-19 vaccination on circulating anti-RBD IgG and IgA antibody levels in individuals post-SARS-CoV-2 infection. Bar graphs (**A**,**C**,**E**,**G**) showing the levels (~ng/mL) of circulating anti-RBD or -NP IgG or IgA antibodies pre-vaccination (the last sample collected before vaccination), post-vaccination (the first sample collected after vaccination), and at the end of sample collection (the last sample collected per individual) were generated, and statistical significance was assessed with the Wilcoxon matched pairs signed rank test. Trajectories of circulating anti-RBD IgG and IgA (**B**,**D**) and anti-N IgG and IgA (**F**,**H**) over 8 separate visits, indicated by color, with Loess regression showing the average trend line (red) and 95% CI of individuals PCR-positive for SARS-CoV-2 (n = 12). Each individual’s first dose of the COVID-19 vaccine series is set as time point 0, with the vertical black line (x = 0) differentiating between pre- (left of x = 0) versus post-vaccination (right of x = 0) time points. The average slope of all time points after vaccination (p_slope after vax)_ is calculated using the Theil–Sen slope estimator, and statistical significance is assessed with the Wilcoxon signed rank test (see methods). *p* > 0.1234 (non-significant), *p* < 0.0021 (**), *p* < 0.0002 (***). Dashed lines represent the LOD of the BU ELISA (LOD = ~1.4 ng/mL).

Wilcoxon matched pair signed rank tests were used to determine statistically significant differences in circulating antibody levels between specific visits (Figure 1A,B and Figure 2A,C,E,G). A two-sided *p*-value of <0.05 was considered statistically significant.

#### 2.8.3. Calculation of Correlation Between Circulating Anti-SARS-CoV-2 Antibodies and Neutralization Activity

Spearman’s rank correlation tests were used to determine statistically significant correlations present between circulating anti-SARS-CoV-2 antibodies and neutralization activity. Spearman’s correlation coefficient (R) ranges from +1 (strong positive correlation) to −1 (strong negative correlation), with R = 0 representing no correlation. *p* < 0.05 was considered a statistically significant association.

## 3. Results

### 3.1. Waning of Anti-Nucleocapsid (N) but Not Anti-Spike Receptor-Binding Domain (RBD) SARS-CoV-2 IgG Plasma Antibody Levels in the Nine Months After Initial SARS-CoV-2 Infection

The serological surveillance of anti-SARS-CoV-2 antibodies facilitates the tracking of infection events and vaccine-induced immune responses within and between communities [30,31]. The mRNA COVID-19 vaccines widely used in the United States elicit immune responses to the SARS-CoV-2 spike protein [32]; therefore, nucleocapsid-specific antibody levels have been used to determine infectious events [33]. A differential decay of anti-N versus anti-spike RBD antibodies after SARS-CoV-2 infection was reported previously [16,34,35,36,37]. We sought to confirm these findings using our highly sensitive BU ELISA protocol [28] and to determine if the antibody trajectories to the N and S proteins vary in vaccine-naïve individuals infected at the start of the pandemic (spring 2020). The analysis of circulating anti-SARS-CoV-2 antibody levels via commercially available ELISAs risks higher false-negative rates because of relatively high limits of detection (LODs) [25,38]. To overcome this limitation, we used our highly sensitive BU ELISA (LOD = 1.4 ng/mL) to assess circulating antibody levels > 1-month PSO. Anti-N and anti-RBD IgG antibodies were measured from 46 individuals with PCR-confirmed SARS-CoV-2 infections at the time of enrollment (Table 1). Antibody levels were measured starting at acute illness and continued up to nine months PSO, resulting in an additional 3–4 blood draws per individual (4–5 samples total). To investigate the trends of antibody longevity in circulation PSO, the average slopes of circulating anti-RBD and anti-N antibodies were calculated as described in the methods. Non-parametric analyses were used to determine if the calculated average slopes were significantly different from slope = 0. All samples collected here pre-date vaccine availability in the United States and therefore represent infection-only, vaccine-naïve individuals. Plasma anti-N IgG levels significantly decreased (*p* = 0.0032) between the first and second blood draws (7–211 days between visits, median 68 days) and continued to decline significantly between the second and third blood draws (*p* = 0.0077) (11–151 days between visits, median 77 days) (Figure 1A), showing an early and progressive decline in anti-N antibodies in the initial months after infection. This trend was not observed for circulating anti-RBD IgG (*p* = 0.6226, visits 1–2; *p* = 0.2192, visits 2–3); instead, mean anti-RBD IgG levels remained stable, with no significant waning, during the first nine months PSO (Figure 1B). The non-parametric analysis of changes in the average slope amongst all individuals revealed a significant decline in anti-N IgG levels (*p* = 0.0073) but not anti-RBD IgG (*p* = 0.477) (Figure 1C,D; individual participant graphs shown in Supplementary Figure S1). These findings confirm previous reports of a faster decline in anti-N IgG levels compared to anti-RBD in a vaccine-naïve cohort [37,39].

### 3.2. Vaccine Recipients That Were Infected with SARS-CoV-2 an Average of Eight Months Prior Exhibit Stable Anti-RBD IgG and IgA Antibody Levels for More than a Year Post-Vaccination

The durability of SARS-CoV-2 anti-N and anti-RBD IgG antibodies nine months to one year after SARS-CoV-2 infection is well established [10,11,34,40,41]. However, the longevity of anti-N antibodies and vaccine-induced antibody stability, one year or more post-infection or vaccination, respectively, in individuals with a history of SARS-CoV-2 infection requires further examination. To address this, we assessed the long-term stability of anti-N and anti-RBD IgG antibody levels amongst a subset of the aforementioned cohort who received a COVID-19 mRNA vaccine 6–11 months after their first SARS-CoV-2 infection (after visit 4–5 of Figure 1). Additionally, SARS-CoV-2 can establish upper respiratory tract infections, resulting in the production of IgA antibodies, which have been shown to play a dominant role in early protection from infection [12,42]; therefore, we measured the levels of anti-N and anti-RBD IgA antibodies as well. Of the 46 individuals initially enrolled, 12 donated plasma samples for an additional 12 or more months post-vaccination (median, 24.3 months PSO) (7–8 samples total). All 12 individuals received the primary vaccine series, and 10/12 (83%) received a COVID-19 booster. The majority of individuals received the Moderna vaccine for both shots in the primary vaccine series, as well as the booster (Supplemental Table S1). When examining the overall change in average antibody levels pre- and post-vaccination in our cohort, we found that average plasma anti-RBD IgG levels significantly increased (*p* = 0.0034) after vaccination and remained elevated until the last collection time point. Circulating anti-RBD IgG levels were significantly higher at the last time point collected compared to immediately pre-vaccination (*p* = 0.0005) (Figure 2A), a time span that averaged 16 months. Overall, in this cohort, the new vaccine-induced antibodies were sustained for at least 12 months in circulation. When average antibody level slopes after vaccination were analyzed per individual, the same result was observed, with no significant decline (*p* = 0.7646) noted in the circulating levels of plasma anti-RBD IgG levels in the year post-vaccination (Figure 2B). The average slopes of circulating anti-RBD and anti-N antibodies after vaccination were calculated as described in the methods, and non-parametric analyses were used to determine if the calculated average slopes were significantly different from slope = 0. A parallel analysis of anti-RBD IgA levels was performed, and a significant increase in plasma IgA levels between pre- and post-vaccination time points was also observed (*p* = 0.0005) (Figure 2C). Although the levels of circulating IgA remained significantly higher at the final collection time point compared to pre-vaccination levels (*p* = 0.0005), there was a small but statistically significant decline (*p* = 0.01855) that was noted in the average slope of IgA levels during the year post-vaccination per individual (Figure 2D).

Anti-N levels are influenced by SARS-CoV-2 infection but not by vaccine administration [38,40]. To verify that the elevation in circulating anti-RBD IgG and IgA levels was attributable to the impact of mRNA vaccination, anti-N IgG and IgA levels were assessed in the same manner as anti-RBD IgG and IgA. Anti-N IgG significantly decreased pre-vaccination (Figure 1A,C); later time points show that anti-N IgG and IgA levels did not significantly increase from pre- to post-vaccination time points (IgG *p* = 0.0537; IgA *p* > 0.9999), and antibody levels measured at the final collection time point were not higher than those collected pre-vaccination (IgG *p* = 0.8457, IgA *p* = 0.5469) (Figure 2E,G). After the initial decline in the average slope for all individuals during the first 6 months PSO (pre-vaccination), both IgG and IgA anti-N levels plateaued in the year post-vaccination (Figure 2F,H). Further, to confirm that the increase in anti-RBD IgG and IgA was not due to a post-vaccine increase in total IgG and IgA, we also measured circulating IgG and IgA levels for Cytomegalovirus (CMV), Epstein–Barr virus (EBV), and the respiratory virus influenza H1N1 Hemagglutinin subunit. No significant changes in antibody levels for any of these three viruses over time were observed (Supplemental Figure S4). Although no subsequent PCR-positive results were provided over the course of our study, large fluctuations observed in anti-N antibody levels were assumed to be reinfection events, with SARS-CoV-2 or an endemic coronavirus strain, as they mostly occurred far after vaccination. Some subjects demonstrated cases of potential reinfection (Figure 1C). However, we aimed to examine the overall trend of anti-N antibodies present in the first 6–9 months PSO, and isolated fluctuations do not influence this (Supplemental Figure S7). Additionally, large fluctuations in anti-RBD levels could be explained as the severe waning of antibody levels, which were boosted and re-elevated immediately following vaccination (Figure 2B,D). As RBD fluctuations represent participant-specific antibody kinetics likely influenced by vaccination, we included all individuals in analyses to determine the overall longevity. To ensure that the overall trend that we observed in anti-RBD or -N antibody longevity was not influenced by suspected reinfections, analyses were performed excluding individuals suspected of being reinfected with SARS-CoV-2, and the significance reported initially was unchanged (Supplemental Figure S6). As a result, all subjects were included in downstream analysis. Overall, these findings confirm that circulating anti-SARS-CoV-2 RBD IgG levels increase and remain elevated for at least one-year post-vaccination, while anti-RBD IgA levels show signs of decline while remaining significantly elevated post-vaccination. These results further inform the long-term trajectories of anti-SARS-CoV-2 IgG and IgA antibodies in hybrid immune individuals.

### 3.3. Temporal Trajectories of Plasma Neutralization Activity Against SARS-CoV-2 Are Analogous, Overall, to Trends Observed in Circulating Anti-RBD IgG and IgA Levels

**Figure 3:**
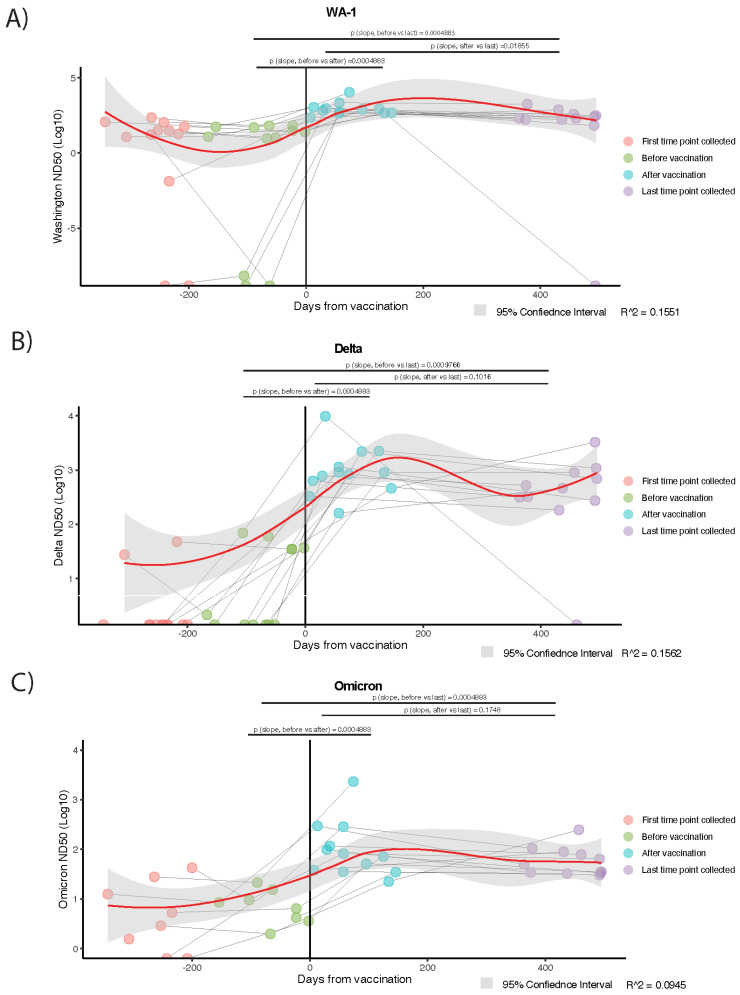
Neutralization activity of plasma from hybrid immune individuals assessed against Washington (WA-1), Delta, and Omicron variants. Fifty percent neutralization dilution (ND_50_) for each individual (n = 12) measured in plasma samples from 3 to 4 distinct time points as indicated by color. Loess regression shows average ND_50_ trend lines (red) with 95% CI for (**A**) Washington (Wuhan), (**B**) Delta, and (**C**) Omicron variants. Shown above each graph are the average slopes of time points spanning from immediately before vaccination to the last time point collected (p_slope, before vs. last_); time points after vaccination to the last time point collected (p_slope, after vs. last_); and the time point immediately before versus after vaccination (p_slope, before vs. after_). Slopes are calculated using the Theil–Sen slope estimator, and statistical significance is assessed with the Wilcoxon signed rank test.

Noting the longevity of the anti-RBD IgG and IgA antibodies in the year following the vaccination of previously SARS-CoV-2 infected individuals, we next examined if viral neutralization activity would match these findings. Neutralization activity against the ancestral SARS-CoV-2 strain (WA-1) and two variants (Delta and Omicron/BA.1) was assessed via neutralization assays using plasma from the same 12 individuals tested in Figure 2. Average slopes for variant-specific neutralization activity were calculated as described in the Methods Section. Deviation from a slope = 0 was used to assess statistical significance using the Wilcoxon signed rank test. All individuals demonstrated a significant increase in neutralizing activity between the pre- and post-vaccination time points for all three variants tested (Figure 3A–C, Supplemental Figure S3). Neutralization activity against the WA-1 strain significantly increased from pre-vaccination (“before vaccination”) to post-vaccination (“after vaccination”) for all individuals but experienced a significant decline in neutralization activity in the year post-vaccination (*p* = 0.01855) (Figure 3A). Despite this decline, neutralization activity was significantly higher at the fourth time point (“last time point collected”) compared to the second time point (“before vaccination”) (*p* = 0.0004883), showing a sustained neutralization ‘footprint’ from the vaccination one year later. Significant increases in neutralization activity were similarly observed against the Delta (*p* = 0.00009766) (Figure 3B) and Omicron (*p* = 0.0004883) (Figure 3C) variants for all individuals post-vaccination (“after vaccination”) as compared with pre-vaccination (“before vaccination”); however, unlike WA-1, there was no significant decline in the neutralization activity observed against either of the other variants in the year post-vaccination time point (Delta *p* = 0.1016; Omi *p* = 0.1748). In sum, we show that neutralization activity against two major variants of concern (VOCs) (WA-1 and Delta) in circulation at the time of this study, as well as the emerging Omicron BA.1 variant, did significantly increase with vaccination in the hybrid immune cohort. Additionally, this neutralization activity was maintained for two (Delta and Omicron) of the three VOCs in the year post-vaccination (Figure 3).

We also examined the relationship between the magnitude of viral neutralization activity and plasma anti-SARS-CoV-2 RBD IgG and IgA levels in individual cohort participants over time. Spearman’s rank correlation tests were performed between virus-specific neutralization activity and anti-RBD IgG or IgA levels, respectively (Supplemental Figure S5) (see Methods). Overall, a strong positive correlation was found between circulating anti-RBD IgG levels and neutralization activity against the WA-1, Delta, and Omicron variants for the twelve individuals during the sample collection period. Additionally, both neutralization activity and circulating anti-RBD levels were increased post-vaccination. A strong positive correlation was also found between circulating anti-RBD IgA levels and neutralization activity against the three variants overall, with both neutralization activity and circulating antibody levels boosted post-vaccination.

## 4. Discussion

This study examines the long-term dynamics of circulating anti-SARS-CoV-2 N and spike RBD IgG and IgA antibodies, as well as viral neutralization activity, over two years PSO—one year post-vaccination—in a cohort of individuals infected with SARS-CoV-2 at the start of the pandemic in the spring of 2020. To our knowledge, this is one of the first studies to document the presence of low-level anti-IgG antibodies in circulation two years PSO, as earlier studies demonstrated difficulties in retroactive detection caused by lower methodological sensitivity leading to premature false negatives [25,38]. As SARS-CoV-2 infection appeared to have elicited robust antibody responses, plasma anti-RBD IgG antibodies, which can prevent viral entry into host cells, demonstrated stability for over nine months PSO (Figure 1A). Following mRNA vaccination, circulating anti-RBD IgG and IgA levels significantly increased, with no significant waning observed up to a year post-vaccination (Figure 2, Supplemental Figure S2). Additionally, the long-term trajectories of circulating anti-RBD IgG or IgA levels mostly paralleled neutralization activity against three SARS-CoV-2 variants—WA-1, Delta, or Omicron—when plasma was assessed via viral neutralization assays (Figure 3C, Supplemental Figure S3). Taken together, these data underscore that hybrid immunity generates a robust and long-lasting humoral response, which likely contributes to sustained, effective protection from infection and/or poor outcomes from SARS-CoV-2 infection.

Hybrid immunity resulting from infection followed by vaccination led to durable, long-lasting anti-RBD IgG responses in all 12 individuals observed in our study (Figure 2A,B, Supplementary Figure S2). Initial infection with SARS-CoV-2 allows for the establishment of RBD-specific memory B cell populations [43,44], which we predict included many clones that were induced to differentiate into antibody-secreting, long-lived plasma cells (LLPCs) following vaccination. An increase in LLPCs coincides with an increase in the production of anti-RBD IgG and IgA antibodies, replenishing any decline in antibody levels in circulation for months post-vaccination [45,46]. As antibodies targeting the RBD of the spike antigen specifically prevent the entry of the virus into host immune cells, the fact that these antibodies demonstrate such stability and longevity in the year post-vaccination (two years PSO) is of great significance as these results add to the current body of evidence supporting a year-long timeframe of protection from SARS-CoV-2 infection [15,17,21,24,42,47,48,49,50]. Investigation into the quantity and longevity of RBD-specific memory B cell populations and LLPCs is worthy of pursuit to reveal their roles in restoring circulating SARS-CoV-2-specific antibodies.

A recent study reported a lack of SARS-CoV-2-specific plasma cells in the bone marrow niche in individuals with COVID-19 vaccination only or hybrid immune status [51]. This work was groundbreaking in its ability to isolate bone marrow antibody-secreting cells (BM ASCs) from 20 COVID-19-vaccinated individuals with a bone marrow aspirate collection spanning from 2020 to 2022. BM ASCs were identified via flow cytometry as CD138+CD38+IgD-cells and SARS-CoV-2-specific IgG production verified via enzyme-linked immunosorbent (ELISpot) assays. Additionally, the findings were further made robust by comparing the “novel” SARS-CoV-2-specific BM ASCs to known established populations of tetanus and influenza populations. The authors speculated that the lack of SARS-CoV-2-specific BM ASCs found in their cohort was due to an inability to develop memory B cells that then establish effector functions in the bone marrow, leading to a lack of long-term antibody secretion into the circulation. These findings are incongruent with our results, showing the durability and presence of anti-RBD IgG and IgA antibodies over six months PSO and up to a year post-vaccination in plasma (Figure 1B and Figure 2A,B,E,F). Others have also reported the longevity of neutralizing antibodies in hybrid immune individuals over a year post-COVID-19 symptom onset [23,52], including findings from another group sampling human bone marrow aspirates that reported the generation of de novo spike-specific B cell responses in germinal centers following Omicron booster administration [43]. The authors acknowledged a lack of variety in self-reported disease severity in their cohort, understanding that symptom severity may influence the formation and durability of the memory cell responses. In particular, the limited number of hybrid immune individuals studied may explain why these results differed from groups reporting SARS-CoV-2 memory B cell responses in hybrid immune individuals [19,23,52].

We found that anti-N IgG antibodies wane earlier than anti-RBD IgG antibodies after SARS-CoV-2 infection (Figure 1). We speculate that the early decline of anti-N antibodies observed reflects a predominance of anti-N-secreting plasmablasts over LLPCs. In cases of mild disease, a lower viral load may limit N-antigen availability to CD4+ Tfh cells, which play a crucial role in influencing memory B cell differentiation into either LLPCs or short-lived plasmablasts [53]. This may reduce N-specific memory B cell differentiation in the germinal center (GC) response [54]. As most of our cohort represent mild COVID-19 disease (n = 33/46 at six months PSO; n = 9/12 at two years PSO; outpatient care), this may explain the early significant decay observed in the initial six months PSO (Figure 1). Others reported significantly lower anti-N IgG levels in cases of mild COVID-19 disease, further supporting this line of thought [16]. In contrast, anti-RBD IgG levels exhibited no significant waning in the year PSO or post-vaccination. One explanation for these results is the increased stability of the pre-fusion spike protein expressing RBD, which may allow for a longer-lived antigen to enhance the affinity maturation process and result in a more robust and subsequently long-lasting anti-RBD IgG response from memory B cells and LLPCs as compared with N protein [55,56,57]. To our knowledge, there has been no extensive investigation into the long-term stability of the N protein, which may impact the affinity maturation process and result in anti-N antibody responses of varying durability. Further exploration into the differences in N and RBD antigen durability and their roles in the GC response and LLPC generation is warranted to better understand the mechanisms driving antibody stability over time.

The relationship between the longevity of antibody responses and the type of vaccine administered remains an open question. Several groups in 2022 investigated the differential antibody kinetics that are observed post-administration of the original vaccine series and found that there were high antibody titers elicited for at least 60 days post-Moderna or Pfizer vaccination [58,59]. Compared to other vaccines administered worldwide, these two vaccines showed higher efficacy and durability in protection from infection [60,61]. Our cohort overwhelmingly received the Moderna vaccine throughout the primary vaccine series and the first booster administration (Supplemental Table S1). As a result, our findings are unable to add to this particular discussion.

Concerns were initially expressed about the potentially ephemeral nature of antibody neutralization activity [62,63]; however, we and others have demonstrated that neutralization activity experiences a gradual decline over time as opposed to an immediate significant drop-off [50,64,65]. This activity is then significantly boosted by vaccine administration and maintained for a year post-vaccination (Figure 3A–C). This sustained neutralization activity presumably results from RBD-specific memory B cell differentiation into antibody-secreting LLPCs in secondary lymphoid organs, a crucial known source of protective antibodies that have been shown to be induced upon SARS-CoV-2 infection and boosted with vaccination [45,66]. Although neutralization activity is the primary CoP for SARS-CoV-2 infection, there is an interest in exploring secondary or alternative CoPs with the emergence of additional SARS-CoV-2 variants [20]. Plasma anti-RBD IgG levels have been correlated with neutralization activity [67]; however, data are lacking for longer time frames (>1 year). Shown here, circulating anti-RBD IgG and IgA antibody levels did not significantly differ from the neutralization activity against the WA, Delta, and Omicron variants in the year post-vaccination (Supplementary Table S1). This suggests that, although not a replacement, plasma anti-RBD IgG levels show the potential to serve as a useful secondary CoP. Since our data show that anti-RBD IgG levels do not significantly decline in the year post-vaccination, while IgA levels do (Figure 2), we predict that anti-RBD IgG levels may serve as a more durable and reliable CoP in the long term.

Studies emerging early in the pandemic determined that an increased number of comorbidities (e.g., obesity, diabetes, and chronic obstructive pulmonary diseases) were associated with an increased risk of SARS-CoV-2 infection and severe COVID-19 disease [68,69,70]. While we acknowledge that comorbidities present an important point of discussion when considering their influence on antibody production and longevity, no significant correlation between the number or type of comorbidities and antibody levels in this cohort was found.

Our findings in this work reinforce the existing body of evidence demonstrating the long-term stability of plasma anti-RBD IgG levels, which we found to be stable for at least one year post-vaccination. Further studies are needed to quantify RBD- or N-specific memory B cell and LLPC populations, in addition to exploring whether heterologous stimulation results in similar long-term immunity. Additionally, understanding the roles of RBD and N antigens in the GC response will provide critical insights into the differences in antibody dynamics observed here and by others. In sum, these results provide new insights into antibody kinetics and the differential waning of immune responses to SARS-CoV-2 and can contribute to datasets informing vaccination strategies and schedules in this post-pandemic era dominated by hybrid immunity.

## Figures and Tables

**Table 1:** Subject demographic and illness features.

	SARS-CoV-2 Positive Individuals
**Total (N)=**	46
**Age in years (median, range)**	44 (25–82)
**Sex (male)**	17 (37%)
**Race**	
Black	2 (15.4%)
Asian	3 (23.1%)
White	37 (80.4%)
Other or not available	5 (10.9%)
Ethnicity (Hispanic)	5 (10.9%)
**Illness features**	
Hospitalized	13 (28.2%)
**Length of hospitalization**	
Median (range)	41 days (5–71)
ICU status (YES)	9 (19.6%)
**Average follow-up time after symptom onset (days)**	
Last sample > 30 and ≤90 days	6 (46.2%)
>90 and ≤180 days	4 (8.7%)
>180 and ≤450 days	19 (41.3%)
>550 days	17 (37%)
**Medical comorbidities**	
Lung disease	2 (6.1%)
Autoimmune disease	6 (46.2%)
Immunosuppressed	10 (21.7%)
Hypertension	2 (6.1%)

## Data Availability

Data can be found within the article and Supplemental Materials.

## Supplementary Materials

The following supporting information can be downloaded at https://www.mdpi.com/article/10.3390/v17050640/s1, Figure S1: Individual longitudinal plasma anti-RBD and anti-N IgG measurements (n = 46) up to 8 months PSO. Figure S2: Changes in serum IgG and IgA levels against SARS-CoV-2 antigens and other viruses for up to a year post-COVID-19 vaccination. Figure S3: Neutralization activity of hybrid immune plasma against WA-1, Delta, and Omicron variants for up to a year post-COVID-19 vaccination. Figure S4: Changes in serum IgG and IgA levels against CMV, EBV (GP350), and H1N1 (HA) antigens for up to a year post-COVID-19 vaccination (n = 12). Figure S5: Correlation of variant-specific neutralization activity with anti-RBD IgG and IgA levels for two years PSO (n = 12). Figure S6: Assessment of overall changes in circulating anti-SARS-CoV-2 RBD and N IgG and IgA antibodies. Figure S7: Assessment of overall changes in circulating anti-SARS-CoV-2 RBD and N IgG and IgA antibodies up to 8 months PSO. Table S1: Distribution of vaccines administered during the primary one (J&J) or two (Moderna and Pfizer) dose vaccine series and booster.

## Abbreviations

The following abbreviations are used in this manuscript:

SARS-CoV-2	Severe Acute Respiratory Syndrome Coronavirus 2
N	Nucleocapsid
S	Spike
RBD	Receptor-binding domain
EBV	Epstein–Barr virus
CMV	Cytomegalovirus
HA	Hemagglutinin
ND50	50% Neutralization Dose
AU	Arbitrary unit
