# Supplementary material for: Individuals Infected with SARS-CoV-2 Prior to COVID-19 Vaccination Maintain Vaccine-Induced RBD-Specific Antibody Levels and Viral Neutralization Activity for One Year
Source: Viruses. 2025 Apr 29;17(5):640. doi: 10.3390/v17050640 (PMC12115583; doi:10.3390/v17050640)
Supplement: Supplementary file 1 [file viruses-17-00640-s001.zip › viruses-3490643-final supplementary/Supp Fig 7 and Supp Table 1.pdf]

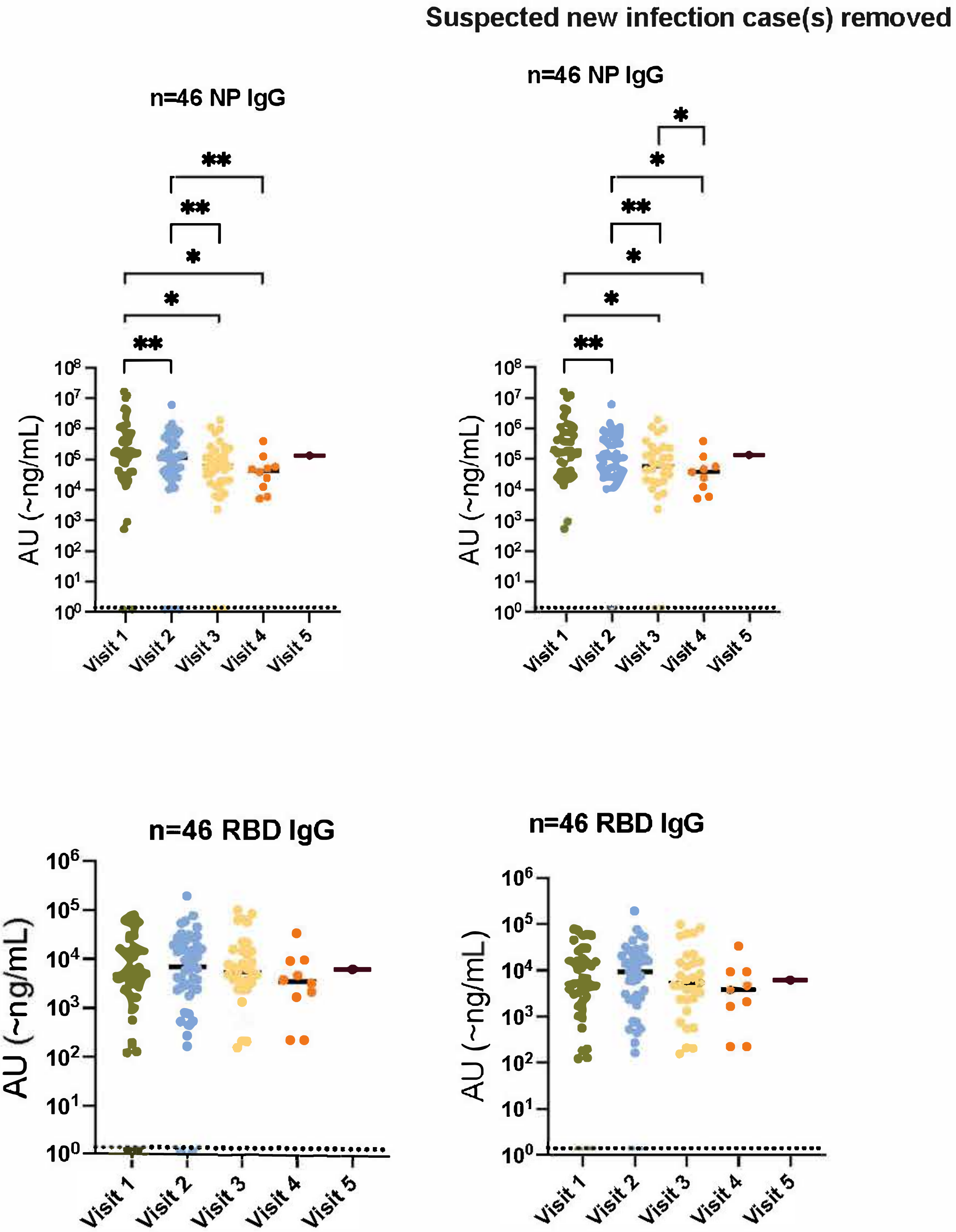

Supplemental Figure 7. Assessment of overall changes in circulating anti-SARS-CoV-2 RBD and N IgG and IgA antibodies up to 8 months PS●. (Left) All twelve individuals displayed in Figure 2 with samples spanning ~2 years PS●. (Right) Samples from Figure 2 with large fluctuations in anti-N antibodies removed. Dashed lines represent te L●D = ~1.4ng/mL.

	Number of Subjects Vaccinated with...		
	Moderna	Pfizer	J&J
1st Vaccination	10	1	1
2nd Vaccination	10	1	N/A
Booster	8	2	0

Supplemental Table 1. Distribution of vaccines adminstered during the primary one (J&J) or two (Moderna and Pfizer) dose vaccine series, and booster.
