# Supplementary figures and images for: Individuals Infected with SARS-CoV-2 Prior to COVID-19 Vaccination Maintain Vaccine-Induced RBD-Specific Antibody Levels and Viral Neutralization Activity for One Year
Source: Viruses. 2025 Apr 29;17(5):640. doi: 10.3390/v17050640 (PMC12115583; doi:10.3390/v17050640)

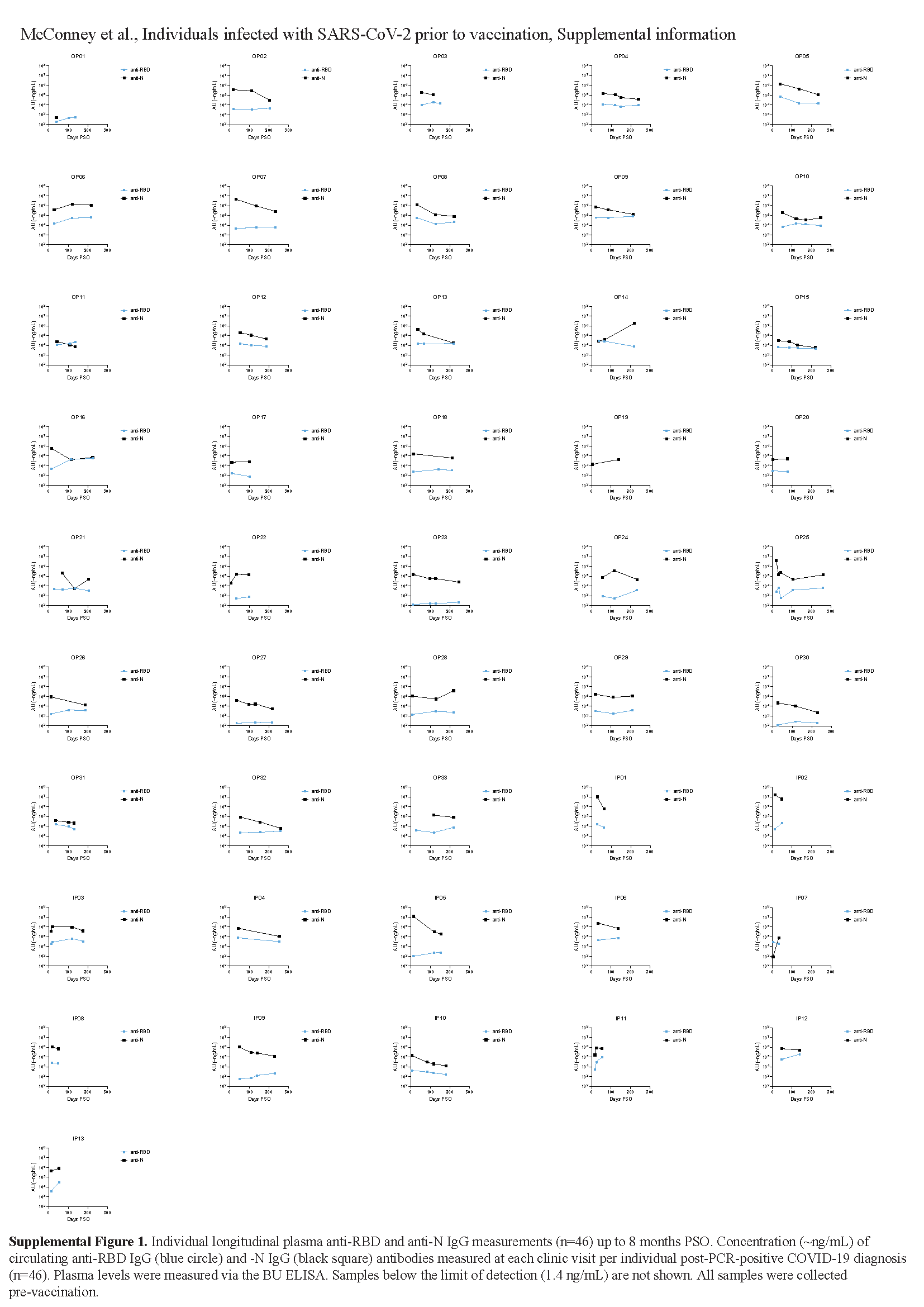

Supplement: Supplementary file 1 [file viruses-17-00640-s001.zip › viruses-3490643-final supplementary/Supplemental Figure 1.tiff]

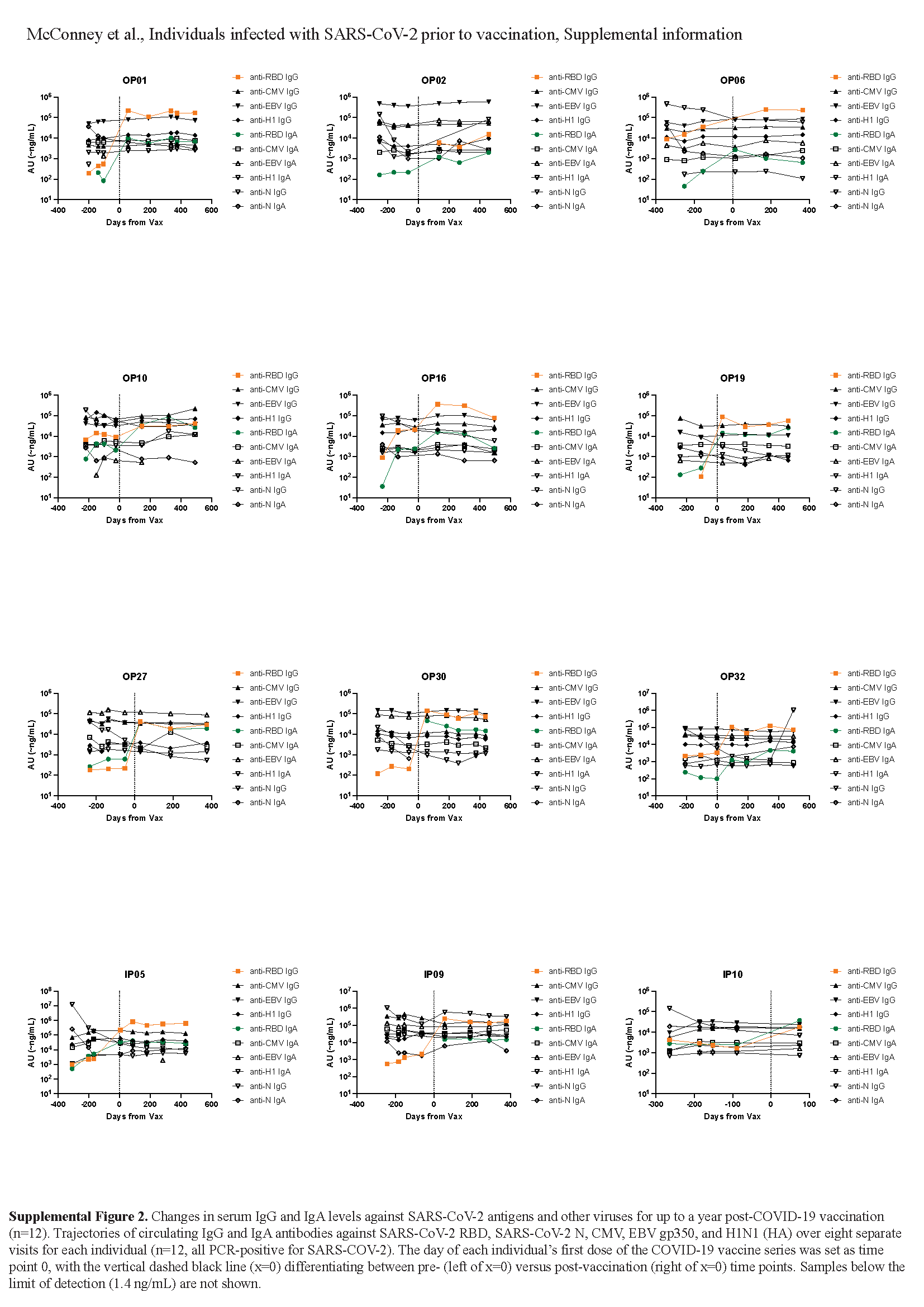

Supplement: Supplementary file 1 [file viruses-17-00640-s001.zip › viruses-3490643-final supplementary/Supplemental Figure 2.tiff]

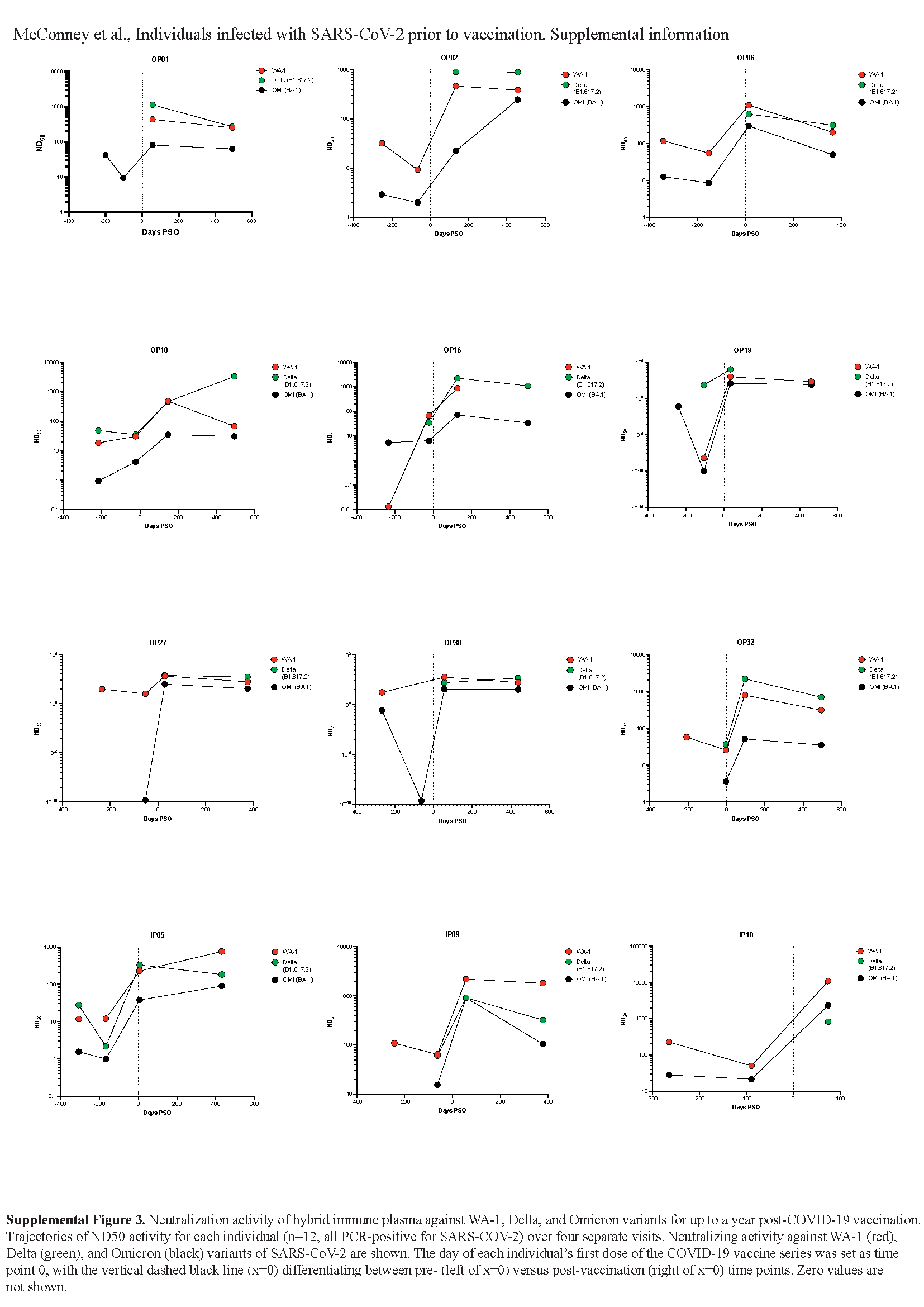

Supplement: Supplementary file 1 [file viruses-17-00640-s001.zip › viruses-3490643-final supplementary/Supplemental Figure 3.tiff]

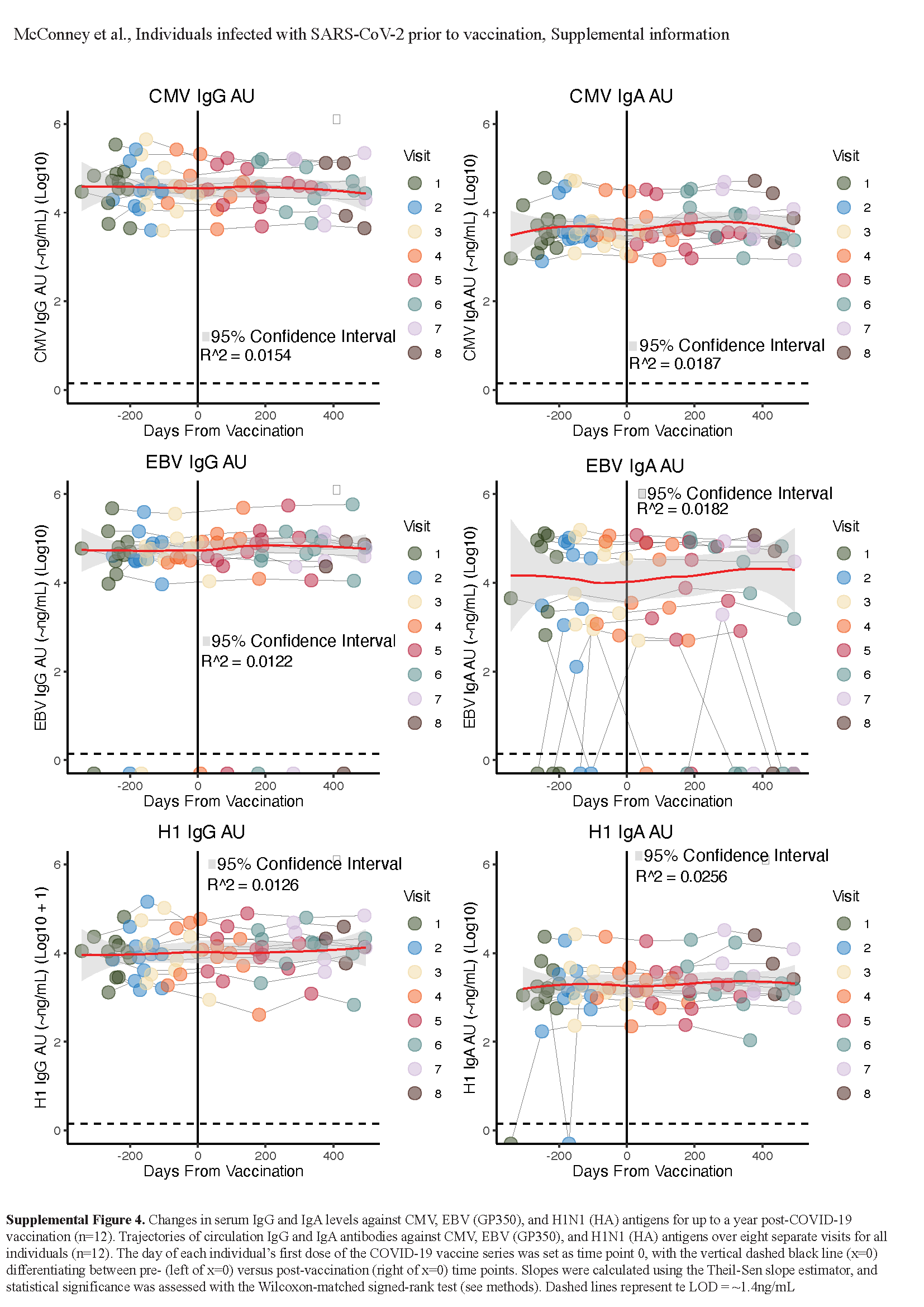

Supplement: Supplementary file 1 [file viruses-17-00640-s001.zip › viruses-3490643-final supplementary/Supplemental Figure 4.tiff]

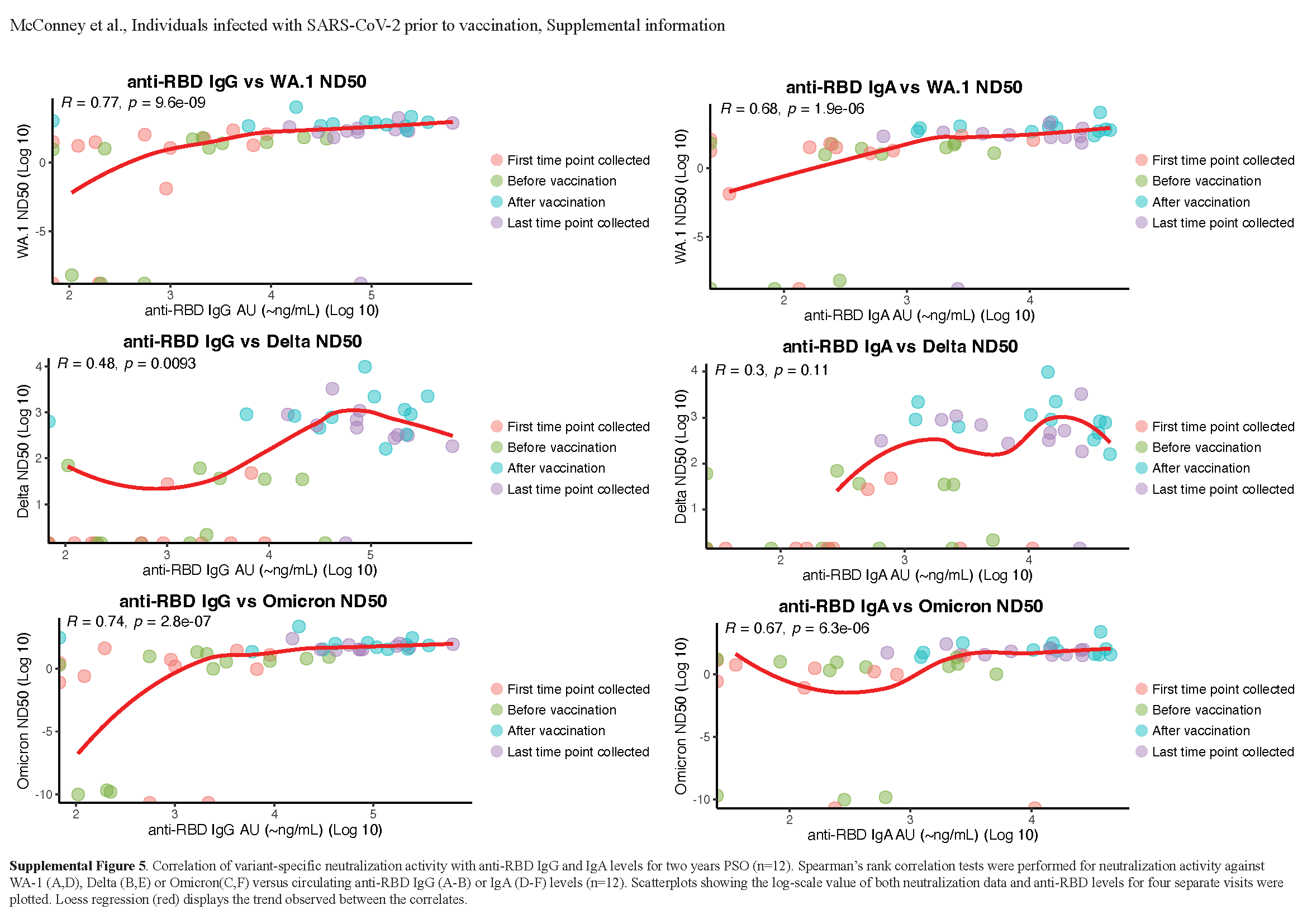

Supplement: Supplementary file 1 [file viruses-17-00640-s001.zip › viruses-3490643-final supplementary/Supplemental Figure 5.tiff]

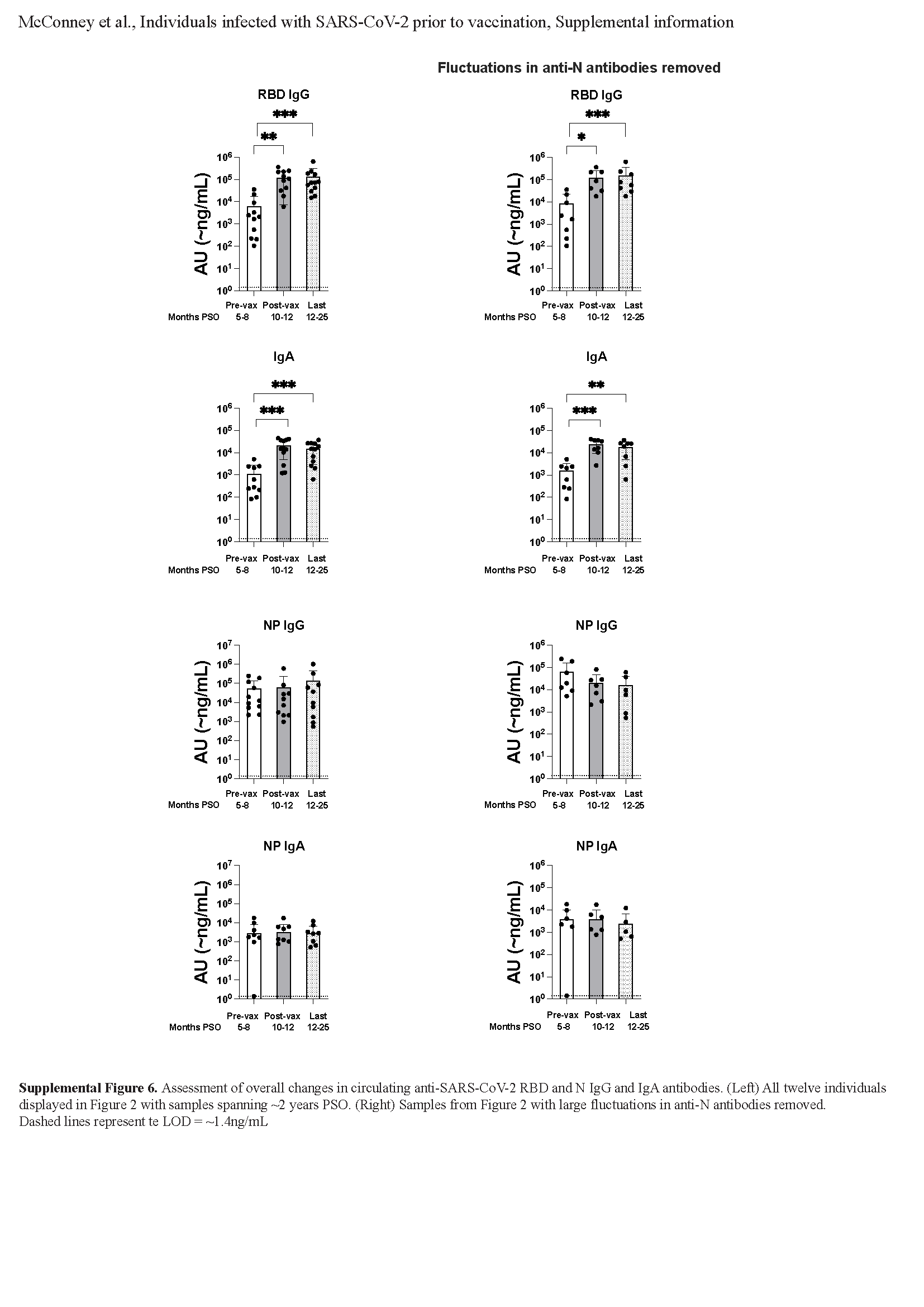

Supplement: Supplementary file 1 [file viruses-17-00640-s001.zip › viruses-3490643-final supplementary/Supplemental Figure 6.tiff]
